# Incarceration's lingering health effects on Black men: impacts persist into retirement

**DOI:** 10.3934/publichealth.2024026

**Published:** 2024-04-16

**Authors:** Shervin Assari

**Affiliations:** Departments of Urban Public Health, Internal Medicine, and Family Medicine, Charles R. Drew University of Medicine and Science, Los Angeles, CA, USA

**Keywords:** incarceration, retirement, social determinants, ethnic groups, health equity

## Abstract

**Background:**

The unique challenges Black men face within the criminal justice system underscore structural and systemic factors driving widespread inequalities. The long-term effects of these challenges on economic, health, and social outcomes as individuals transition to retirement remain poorly understood, highlighting a critical gap in our knowledge of life trajectories long after justice system involvement.

**Objectives:**

This study investigated the enduring health impacts of incarceration on Black men, particularly focusing on the transition into retirement. It aimed to explore the influence of race and gender on experiences of incarceration before age 50, and how such experiences affected self-rated health during the retirement transition.

**Methods:**

Utilizing data from the Health and Retirement Study, which followed individuals aged 50–59 for up to thirty years, this research examined the interplay of race, gender, incarceration history, and self-rated health during the retirement transition. Logistic regression and path modeling were employed for data analysis.

**Results:**

Logistic regression results indicated that being Black, male, and having lower educational attainment significantly increased the likelihood of experiencing incarceration before the age of 50 (p < 0.05). This suggests that Black men with lower levels of education are at the greatest risk of incarceration. The path model revealed a correlation between incarceration experiences before age 50 and poorer self-rated health at the time of retirement.

**Conclusion:**

The findings highlighted the disproportionately high risk of incarceration among Black men, especially those with lower educational attainment, and its persistent negative impacts on health decades later, including during the transition into retirement. Addressing structural racism and the mass incarceration of Black men is crucial for achieving racial health equity as individuals retire.

## 1. Introduction

Structural racism [1], a pervasive force underlying economic and health disparities of Black men, manifests prominently in various sectors, with the criminal justice system standing out as a focal point [2]. The historical and deeply ingrained racial disparities in imprisonment [3], particularly affecting Black men [4], epitomize the core element of systemic racism [5]. Over centuries, Black men have consistently borne the highest imprisonment rates compared to any other racial or gender group [6]–[8].

This disparity is not a consequence of behavioral differences but rather a stark marker of the deeply rooted racism permeating American society across institutions [9],[10]. From poor education opportunities in impoverished neighborhoods to the well-described school-to-prison pipeline [11], Black children face a multitude of challenges. These challenges, including higher dropout rates, increased discipline, exposure to violence, and residing in high-crime neighborhoods, create a milieu that propels them towards incarceration. Racial residential segregation further limits employment opportunities, increasing the risk of involvement in criminal activities. Sentencing disparities are also well-described in the literature [12],[13].

Black men who come into contact with the criminal justice system not only grapple with the immediate consequences of incarceration but also confront enduring chains of economic and health inequalities that persist for decades [14]. The aftermath of imprisonment often leads to diminished employment prospects, increased engagement in risky behaviors, and heightened vulnerability to mental health issues [15]. Social isolation, a common consequence, further exacerbates health hazards [16].

Investigating how imprisonment shapes the long-term economic and health opportunities of Black men in the United States [17], in comparison to other race and gender intersectional groups, is crucial [18]. The disproportionate incarceration rates, symptomatic of racial bias, serve as a catalyst for a cascade of challenges hindering economic mobility and access to the labor market, economic opportunities, and quality healthcare [19]. Examining these issues through a racial justice lens emphasizes the imperative for systemic reforms that address the root causes of this pervasive cycle [20], liberating Black men from a life of enduring racism-related disadvantage [21].

Despite the evident impact of incarceration on individuals [22], there exists a dearth of comprehensive studies exploring its connection to various long-term outcomes, encompassing behavioral, health, social, psychological, and economic dimensions [23]. Additionally, limited research focuses on the effects of this history on middle-aged and older adults [24]. While some cross-sectional studies exist, a scarcity of long-term longitudinal studies, particularly on a national scale, underscores the need for more robust research [25],[26]. Furthermore, employing an intersectionality framework to compare race and gender groups is essential for understanding the unique experiences of Black men in these complex and interconnected realms [27].

Intersectionality theory [28]–[30], pioneered by Kimberlé Crenshaw in 1989, offers a comprehensive framework to understand the intricate interplay of multiple social identities and systems of oppression. When applied to the experiences of Black men [31],[32], intersectionality recognizes that their lives are shaped by the convergence of various social categories, including race, gender, class, and more [33]. This perspective acknowledges that the discrimination faced by Black men is not solely based on their race but is profoundly influenced by their gender as well [34]. Consequently, Black men navigate a unique intersection of racism and sexism, leading to distinct challenges and stereotypes that differ from those faced by Black women or White men. The theory extends to economic disparities, considering the intersection of race and class, which can compound challenges for Black men, particularly those from low-income backgrounds [35]–[37].

In the criminal justice system, due to the mass incarceration of Black men, the intersection of race and gender has created a distinct set of challenges, including racial profiling, discriminatory policing, and harsher sentencing [13],[38],[39]. Health outcomes, educational experiences, and media representations are also intricately shaped by the intersections of race and gender, highlighting the need for a nuanced understanding of the multifaceted factors that influence the lives of Black men [40]. Recognizing and addressing these intersecting systems of oppression is crucial for developing comprehensive strategies to tackle the complexities of their social reality and fostering a more equitable society [41].

## 2. Aims

Informed by the intersectionality theory [28]–[30], this study utilized data from the Health and Retirement Study to assess whether, among people transitioning into retirement, Black men are more likely to have experienced incarceration before age 50. We hypothesized that being Black and being male would be associated with increased odds of past incarceration compared to other race and gender groups. We also tested whether being incarcerated before age 15 is associated with worse health. We hypothesized that a positive history of incarceration will be correlated with poorer health as individuals transition from middle and older adulthood into retirement.

## 3. Methods

### 3.1. Design and setting

Data were obtained from the first 15 waves of the Health and Retirement Study (HRS) [42] conducted from 1992 to 2020. We used the 2020 RAND HRS data set [43] that was publicly released in March 2023. The HRS is a state-of-the-art longitudinal study of retirement transitions in the United States, with biannual repeated measurements. The study recruited and followed a nationally representative sample of middle-aged and older adults (aged 50–59 years at baseline). The HRS study collected extensive data on various aspects of participants, including demographic, socioeconomic, social, psychological, economic, employment, and health data, as well as health behaviors and health service utilization. HRS data have also measured a wide range of data related to retirement including retirement timing [44]. Data were collected through telephone or face-to-face interviews, and proxy interviews were used for participants who were unavailable. Detailed information on the HRS design, measures, sample, and sampling processes can be found elsewhere, and a brief overview is provided here.

### 3.2. Sample and sampling

The HRS used a national area probability sample to recruit participants aged 50 to 59 at baseline. For the current analysis, only the core (primary) sample recruited in 1992 was included to offer the longest follow-up period. All participants in our HRS sample were born between 1931 and 1941, and the sample reflects all middle-aged and older adults aged 50–59 residing in US households in the year 1992 (baseline = wave 1).

### 3.3. Analytical sample

The analytical sample for this study included Americans aged 50 and above who identified as Black or white. Individuals from other racial groups were not included in the analysis. All participants from the HRS core sample were eligible for analysis regardless of the duration of follow-up or the time of mortality. The analytical sample comprised 2529 non-retired participants at baseline, followed for up to 30 years, who were either white or Black, and had data available on our study variables, including incarceration history before the age of 50 (life history). Data from partners or spouses were also collected in the HRS, but only data from the participants themselves were utilized in this study.

### 3.4. Measures

*Predictor*. To measure incarceration history, participants were asked whether they had been imprisoned before the age of 50. The question read as: Have you ever been in a jail, prison, or a detention center for more than 3 days? Responses were yes or no [45].

*Outcome*. Individuals reported their self-rated health every two years from 1992 to 2018. The specific question was: “Would you say your health is excellent, very good, good, fair, or poor?” Responses were coded as 1 reflecting poor health and 0 as better health. Self-rated health is shown to be a strong, valid, and reliable predictor of mortality, even when clinical and paraclinical indicators are controlled [46]. We used cluster analysis to generate two clusters, based on up to 30 years of self-rated health data: those who consistently had poor health, and those who consistently had good health.

*Age*. Age (in years) was treated as a continuous variable, calculated based on the number of years since birth.

*Educational Attainment*. Years of schooling were self-reported. For the first variable, educational attainment was measured as the number of years of schooling, treated as a continuous variable. Education was self-reported at baseline in 1992.

*Gender*. Gender was a dichotomous variable that was coded as 0 for female and 1 for male.

*Race*. Self-identified race was a dichotomous variable that was coded as 0 for white and 1 for Black.

### 3.5. Data analysis

Data analysis was conducted using SPSS 25.0 (IBM Corporation, Armonk, NY, US). Univariate analyses reported means and standard deviations (SD) for continuous variables and absolute and relative frequencies (n and %) for categorical variables. Pearson correlation tests were utilized for bivariate correlations. Additionally, bivariate analyses included comparing individuals with and without a history of incarceration across study variables using chi-square tests for categorical variables and independent samples *t*-tests for continuous variables. In the multivariable analysis phase, logistic regression and path modeling were employed. Two logistic regression models were developed: the first model used incarceration history as the predictor, with self-rated health over the follow-up period as the outcome variable, adjusting for race, age, and gender as control variables. The second logistic regression model inverted the focus, treating incarceration history as the outcome variable, with race, gender, and age serving as predictors. A path model was subsequently implemented to explore the hypothesis that race and gender influence the likelihood of incarceration, which in turn affects the age at retirement. Retirement age was hypothesized to predict health status at the time of retirement. This model included health status at study entry as a control variable.

### 3.6. Ethics statement

The HRS study protocol was approved by the University of Michigan Institutional Review Board. All HRS participants signed written consent. The data were collected, restored, managed, and analyzed in a fully anonymous fashion. As we used fully de-identified publicly available data, this study was considered non-human subject research, according to the NIH definition.

## 4. Results

**Figure 1.**
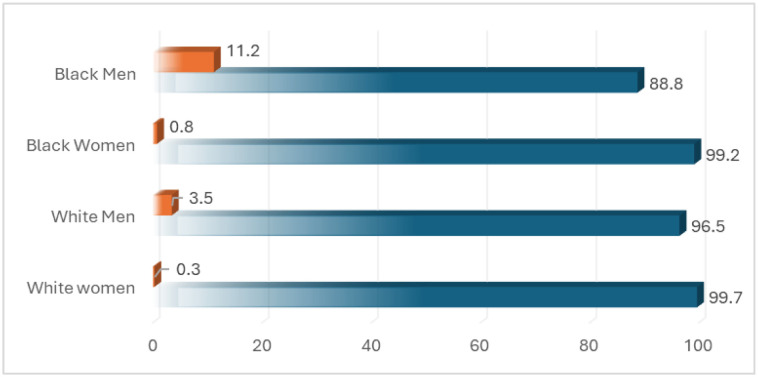
History of incarceration by the intersection of race and gender (the orange color reflects the incarceration rate, and the blue color represents no incarceration history).

Our analysis encompassed 2529 middle-aged and older adults. As illustrated in Table 1 and Figure 1, a history of incarceration was predominantly observed in Black men, with white men following closely (p < 0.05).

**Table 1.** Prevalence of incarceration before the age of 50 in our sample by race and gender interaction (n = 2529).

		Incarceration Hx			
		No		Yes	
		n	%	n	%
White Women	N = 1255	1251	99.7	4	0.3
White Men	N = 911	879	96.5	32	3.5
Black Women	N = 238	236	99.2	2	0.8
Black Men	N = 125	111	88.8	14	11.2

### 4.1. Bivariate analysis

Table 2 compares study variables between two groups differentiated by their incarceration history. It reveals that individuals with a history of incarceration were predominantly male, Black, and reported poorer self-rated health during the follow-up period. Furthermore, these individuals were more likely to experience poverty throughout the 30-year follow-up period.

Table 3 presents the bivariate correlations among study variables. A history of incarceration was found to be associated with being male, identifying as Black, and experiencing poor self-rated health during the follow-up period. Additionally, a history of incarceration showed correlations with poorer self-rated health and living in poverty throughout the 30-year follow-up period.

**Table 2.** Comparison of study variables between individuals with and without incarceration history before age 50.

	Prison -			Prison +		
	n	Mean	SD	n	Mean	SD
Age at baseline*	2477	54.26	2.72	52	53.81	2.65
Baseline self-report of health*	2477	2.16	1.027	52	2.40	1.14
Total of all assets baseline	2477	265,085.22	473,646.75420	52	92,121.0591	109,029.48
Education years	2477	13.02	2.52568	52	11.9423	3.21
% Poverty (0–1)	2477	0.05	0.14	52	0.10	0.19
Race
White	2130	86.0			36	69.2
Black	347	14.0			16	30.8
Gender
Women	1487	60.0			6	11.5
Men	990	40.0			46	88.5
Education
Less than high school	366	14.8			18	34.6
GED	116	4.7			6	11.5
High-school graduate	900	36.3			14	26.9
Some college	524	21.2			6	11.5
College and above	571	23.1			8	15.4
Baseline poverty
Not poor	2356	95.1			48	92.3
Poor	121	4.9			4	7.7
Poverty cluster
Not poor	2098	84.7			35	67.3
Poor	32	1.3			1	1.9
Missing	347	14.0			36	69.2
Poor self-rated health at baseline
Good	1563	63.1			33	63.5
Poor	52	2.1			2	3.8
Missing	862	34.8			17	32.7

**Table 3.** Bivariate correlations between incarceration history and other study variables.

	1	2	3	4	5	6	7	8	9	10	11	12
1 Imprisonment	1	0.15**	−0.03	0.16**	−0.09**	−0.12**	−0.05**	0.04*	0.20**	0.09**	0.02	0.14**
2 Black		1	−0.03	−0.04**	−0.17**	−0.15**	−0.16**	0.17**	0.35**	0.34**	0.20**	0.22**
3 Age			1	0.25**	−0.03*	−0.02	0.10**	−0.04*	−0.08**	−0.04	0.09**	0.02
4 Male				1	0.01	0.05**	0.05**	−0.05**	−0.08**	−0.04**	−0.01	−0.01
5 Married Baseline					1	−0.09**	0.14**	−0.22**	−0.16**	−0.10**	−0.08**	−0.12**
6 Education						1	0.19**	−0.18**	−0.26**	−0.09**	−0.28**	−0.25**
7 Baseline Total Assets							1	−0.09**	−0.14**	−0.05**	−0.16**	−0.14**
8 Baseline Poverty								1	0.48**	0.38**	0.16**	0.13**
9 % Poverty Over Time									1	0.50**	0.26**	0.30**
10 Poverty Class										1	0.13**	0.13**
11 Baseline Self-rated Health											1	0.56**
12 Poor Self-rated Health Over Time												1

Note: *p < 0.5; **p < 0.01.

### 4.2. Logistic regression

Table 4 outlines the factors correlated with a history of incarceration at baseline. The analysis indicates that lower educational attainment, being male, and being Black were significantly associated with increased odds of incarceration history. However, baseline poverty status did not significantly influence the odds of having a history of incarceration before age 50.

**Table 4.** Predictors of incarceration history before age 50 in the population transitioning into retirement.

	aOR	95% C.I. for aOR		Sig.
Age at baseline	0.894	0.799	1.001	0.051
Male	15.724	6.537	37.818	<0.001
Black	2.138	1.079	4.236	0.029
Married	0.418	0.218	0.803	0.009
Education				0.001
Less than high school	Ref.			
GED	1.351	0.491	3.720	0.561
High-school graduate	0.357	0.169	0.758	0.007
Some college	0.256	0.096	0.681	0.006
College and above	0.263	0.107	0.648	0.004
Poverty (baseline)	0.920	0.300	2.819	0.883
Constant	4.885			0.612

Note: aOR = adjusted odds ratio; GED = general educational development.

Table 5 presents the results of our logistic regression analysis, with poor self-rated health throughout the follow-up period as the outcome variable. The data reveal that a history of incarceration is significantly linked to poorer self-rated health during the follow-up.

**Table 5.** Association between incarceration history before age 50 and odds of having poor self-rated health over time and at retirement.

	aOR	95% C.I. for EXP(B)		Sig.
Jailed before 50	1.980	1.102	3.558	0.022
Black	2.614	2.067	3.307	<0.001
Male	0.965	0.796	1.171	0.718
Age at baseline	1.028	0.993	1.064	0.114
Constant	0.059			0.003

Note: Outcome: Poor self-rated health (SRH) over up to a 30-year follow-up period.

### 4.3. Path analysis

**Figure 2.**
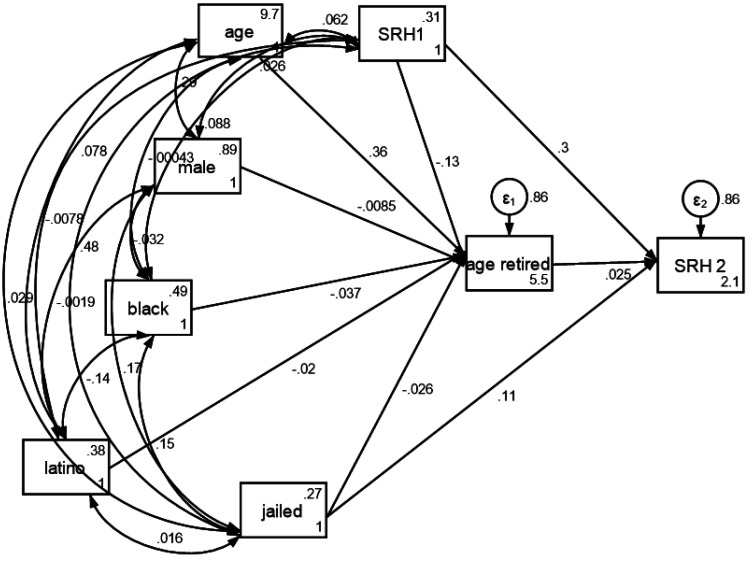
Path model (structural equation model) on the association between incarceration before age 50 and self-rated health at retirement.

As Table 6 and Figure 2 show, incarceration before age 50 was not correlated with age at retirement (p = 0.328), however, it was associated with worse self-rated health at the time of retirement (B = 0.11, p < 0.001). Some other significant associations in our path model included a positive autoregressive association between baseline self-rated health and self-rated health at the time of retirement (B = 0.30, p < 0.001) and positive association between age at retirement and self-rated health at retirement (B = 0.03, p = 0.039).

**Table 6.** Path coefficients in a structural equation model testing the association between incarceration before age 50 and self-rated health at retirement.

Independent Variable	Dependent Variable	B	SE	95% CI		p
Age (Baseline)	Age at Retirement	0.36	0.01	0.33	0.38	0.000
Male	Age at Retirement	−0.01	0.01	−0.03	0.02	0.489
Black	Age at Retirement	−0.04	0.01	−0.06	−0.01	0.005
Latino	Age at Retirement	−0.02	0.01	−0.05	0.01	0.155
Baseline Self-rated Health (Poor)	Age at Retirement	−0.13	0.02	−0.17	−0.09	<0.001
Jailed Before 50	Age at Retirement	−0.03	0.03	−0.08	0.03	0.328
Intercept	Age at Retirement	5.51	0.15	5.22	5.80	<0.001
Age at Retirement	Retirement Self-rated Health	0.03	0.01	0.00	0.05	0.039
Baseline Self-rated Health (Poor)	Retirement Self-rated Health	0.30	0.02	0.26	0.35	<0.001
Jailed Before 50	Retirement Self-rated Health	0.11	0.03	0.05	0.17	<0.001
Intercept	Retirement Self-rated Health	2.13	0.11	1.90	2.35	<0.001

## 5. Discussion

This study validated our hypothesis by illustrating that Black men exhibit the highest prevalence of incarceration before the age of 50. With a prevalence rate of 11%, this figure significantly exceeds those of Black women, white men, and white women. Furthermore, our findings indicate that a history of incarceration is a predictor of poor self-rated health decades later, specifically as individuals approach retirement. Notably, this effect persists beyond the influence of age at the time of retirement or baseline self-rated health, suggesting that the impact of incarceration history on health outcomes extends well into later life stages.

The significant disparity in incarceration rates among Black men compared to other demographic groups highlights the systemic inequalities within the criminal justice system. Moreover, the lasting impact of incarceration on self-rated health underscores the profound and enduring health disparities faced by formerly incarcerated individuals. These findings point to the necessity of addressing both the immediate and long-term health needs of this population, emphasizing the critical role of supportive policies and interventions in mitigating the adverse health effects associated with incarceration history. By understanding the lasting influence of incarceration on health outcomes, stakeholders can better tailor strategies to improve the health and well-being of individuals as they transition into retirement, moving toward a more equitable health landscape for all.

This study unraveled the unique experiences of Black men within the criminal justice system and assessed their subsequent impact on economic, health, and social outcomes during the transition to retirement. By utilizing long-term longitudinal data from the Health and Retirement Study [42],[47],[48], we sought to contribute nuanced insights into the consequences of early incarceration for Black men, a population consistently affected by disproportionate imprisonment rates. Our findings underscored a pronounced disparity, revealing that Black men, particularly before the age of 50, faced a higher likelihood of incarceration compared to other race and gender intersections. This early experience of imprisonment was associated with poor health.

Our results align with existing literature highlighting the adverse effects of incarceration on various life domains of Black men [14]–[16],[22],[23]. The unique contribution of this study lies in its longitudinal long-term follow-up data of groups based on the intersectionality of race and gender, shedding light on the distinct long-term challenges faced by Black men decades after incarceration. This study shows that outcomes of incarceration can be observed long after the experience, elucidating the enduring impact of incarceration on economic, health, and social trajectories into retirement.

Our findings hold significant implications due to the United States having the highest proportion of citizens incarcerated in prisons or jails compared to other Western nations, with an annual admission rate of eleven million people to U.S. jails. The act of imprisonment itself can engender social disadvantages and isolation, which may contribute to a decline in overall health and well-being. Furthermore, individuals involved in the justice system often undergo life experiences that are known to adversely affect health outcomes. Key mechanisms linking imprisonment to future economic and health challenges include limited access to quality healthcare, social isolation from family, inadequate education, heightened risk of homelessness, susceptibility to substance use, unemployment, and exposure to various types of traumas. Consequently, those who have been incarcerated may face a deterioration in their health. Notably, some research suggests that involvement with the criminal justice system may be associated with a phenomenon akin to “premature” or “accelerated” aging [49].

In a landmark analysis of a nationally representative sample of Black men, Assari and colleagues explored the intricate associations between a lifetime history of incarceration, experiences of discrimination, and mental health outcomes in adulthood. The study encompassed 1271 Black men who participated in the National Survey of American Life (NSAL) conducted between 2001 and 2003. The findings revealed significant correlations among incarceration history, depressive symptoms, psychological distress, and everyday discrimination. Utilizing structural equation models (SEMs) with age, education, and income as covariates, the researchers demonstrated that, for Black men, a history of incarceration was fully mediated by everyday discrimination. This mediation was observed in the associations between incarceration history and both depressive symptoms and psychological distress. The study highlighted a crucial insight: Black men with a history of incarceration are likely to encounter future discrimination, thereby increasing their vulnerability to mental health problems. Additionally, the research proposed that dismantling discriminatory practices could potentially mitigate the impact of incarceration on the mental health of Black men, given the observed full mediation. The implications of their findings suggested that policies aimed at reducing preventable incarceration or, at the very least, minimizing subsequent discrimination for those with a history of incarceration, may contribute to an improvement in the mental health outcomes of previously incarcerated African American men. The study underscores the importance of addressing both incarceration rates and discriminatory practices to foster better mental health outcomes within this demographic [45].

The disproportionate rates of imprisonment among Black men serve as a glaring manifestation of structural racism deeply embedded in the fabric of the United States [14]–[16],[22],[23]. The findings reinforce the argument that the criminal justice system, as a key societal institution, plays a pivotal role in perpetuating racial disparities, acting as both a consequence and perpetuator of systemic racism [5]–[8],[38].

### 5.1. Policy implications

Addressing the root causes of racial disparities in incarceration is paramount for achieving economic and social justice. Policy interventions should focus on dismantling structural racism within the criminal justice system, promoting alternatives to incarceration, and implementing measures that facilitate reintegration into society for individuals with a history of imprisonment. Educational and employment opportunities should be bolstered to break the cycle of disadvantage. Policies geared towards criminal justice reform, equitable education, and workforce development are pivotal in undoing the trends identified in this study. Investments in community-based initiatives that address the root causes of incarceration, such as poverty and lack of opportunity, can contribute to breaking the cycle of imprisonment and its associated consequences.

### 5.2. Future direction of research

Future research should delve deeper into the nuanced intersections of race, gender, and age, considering the long-term consequences of imprisonment on specific subgroups within the Black population [50]. A more extensive exploration of the multifaceted factors contributing to post-incarceration outcomes, including community support systems, is warranted. Additionally, research should assess the effectiveness of specific policies aimed at reducing the incarceration of Black men and mitigating the adverse effects of incarceration on Black men.

### 5.3. Limitations

While our study provides valuable insights, it is not without limitations. The reliance on self-reported data poses potential biases, and the sample may not fully capture the diverse experiences within the Black population. Additionally, our findings are based on observational data, limiting causal inferences [51]–[54]. Future research should incorporate a more expansive and diverse data set to enhance the generalizability of our results [55].

## 6. Conclusion

In conclusion, our study highlights the lasting impact of incarceration on the health of Black men, revealing that the adverse health effects of such exposure can persist for decades, becoming particularly evident as they transition into retirement. Structural racism, manifested in the form of disproportionate imprisonment rates, plays a crucial role in creating health disparities among Black men. Therefore, addressing the disparities in incarceration rates and reforming the policing practices in Black communities are essential steps toward breaking the cycle of disadvantage and promoting a more equitable society. By tackling these root causes, we can pave the way for significant improvements in the health and well-being of Black men, ensuring fairer health outcomes for future generations.

## Use of AI tools declaration

The authors declare they have not used Artificial Intelligence (AI) tools in the creation of this article.
